# Phase I/II Prospective Study on Endoscopic Ultrasound‐Guided Hepaticogastrostomy as Primary Drainage for Unresectable Malignant Hilar Biliary Obstruction

**DOI:** 10.1002/deo2.70330

**Published:** 2026-05-04

**Authors:** Tomoki Ogata, Kazuo Hara, Nozomi Okuno, Shin Haba, Takamichi Kuwahara, Shimpei Matsumoto, Hiroki Koda, Keigo Oshiro

**Affiliations:** ^1^ Department of Gastroenterology Aichi Cancer Center Nagoya Japan

**Keywords:** bismuth type IV, endoscopic ultrasound‐guided hepaticogastrostomy, malignant hilar biliary obstruction, phase I/II trial, primary drainage

## Abstract

**Objectives:**

Various drainage methods have been used for unresectable malignant hilar biliary obstruction (MHBO). However, controlling cholangitis and obstructive jaundice is still challenging. We performed a phase I/II study of endoscopic ultrasound‐guided hepaticogastrostomy (EUS‐HGS) as primary drainage for MHBO.

**Methods:**

This prospective Phase I/II study included 20 patients requiring biliary drainage for MHBO recruited from June 2021 to December 2023. The primary endpoint was safety. The secondary endpoints were clinical efficacy, reintervention methods, and frequency.

**Results:**

The types of bile duct stenosis were Bismuth type I/II/IIIa/IIIb/IV; 4/1/5/3/7 cases. Fifteen cases (75%) had cholangitis before the EUS‐HGS was performed. Technical success rate; 100% (20/20), clinical success rate; 75% (15/20). The four cases in which clinical success was not achieved were Bismuth type IV with cholangitis and obstructive jaundice, and all cases required additional drainage for the right bile duct.

Early adverse events were stent migration: one case (severe) and peritonitis: one case (mild).

**Conclusions:**

The safety of EUS‐HGS for the primary drainage of unresectable MHBO is acceptable. Regarding clinical efficacy, based on the results observed in patients with Bismuth type IV obstruction, we acknowledge that further investigation is warranted.

**Trial Registration**: UMIN‐CTR: UMIN000047702.

## 1 Introduction

Unresectable malignant hilar biliary obstruction (MHBO) is caused by bile duct cancer or metastasis from cancers of other organs. Management of cholangitis and obstructive jaundice is extremely important. Endoscopic ultrasound‐guided hepaticogastrostomy (EUS‐HGS) has been developed as a treatment for biliary obstruction and has gained popularity recently, with studies reporting high technical and clinical success rates [1, 2, 3]. Several studies have reported on the usefulness of EUS‐HGS drainage in patients with perihilar cholangiocarcinoma [4, 5, 6, 7]. However, although some of these reports demonstrate the usefulness of EUS‐HGS for MHBO, they were all based on cases in which EUS‐HGS was performed as a rescue procedure. No cases in which EUS‐HGS was performed as the primary biliary drainage procedure have been reported. Therefore, this phase I/II study was designed to evaluate the safety and efficacy of EUS‐HGS as a primary biliary drainage method.

## 2 Methods

This single‐center prospective phase I/II trial was conducted at the Aichi Cancer Center Hospital, Japan. This study included 20 patients who consented to participate between June 2021 and December 2023. The inclusion criteria were as follows: 1) requirement for biliary drainage due to unresectable malignant hilar obstruction, 2) age ≥20 years, and 3) written consent obtained from the patient. The exclusion criteria were as follows: 1) biliary drainage was performed, and 2) patients were deemed inappropriate for this study by the treating physician. A total of 20 patients were assessed for eligibility during the study period. All of these patients met the inclusion criteria and did not meet any exclusion criteria. Therefore, no eligible patients were enrolled, and there were no cases of refusal or withdrawal of consent. In the present study, pathological biopsy of the lesion was performed in all patients except for one case. Pathological tissue diagnosis was obtained in 19 patients, and in all cases, the biopsy was performed from the primary lesion. The methods of tissue acquisition were EUS‐guided tissue acquisition (EUS‐TA) in 12 cases, endoscopic biopsy in six cases for colorectal cancer and gastric cancer, and renal biopsy in one case for kidney cancer. In all patients, biliary drainage was performed after confirmation of the pathological diagnosis. In the one patient in whom a pathological biopsy was not performed, the lesion was clinically diagnosed as hepatocellular carcinoma based on the imaging findings and elevated PIVKA‐II levels; therefore, a biopsy for pathological confirmation was not performed.

In Phase I, the first five patients were enrolled to assess safety. If none of the following adverse events (AEs) occurred within 30 days after the procedure, the study proceeded to Phase II; 1) fever ≥38.5°C lasting more than 7 days, excluding stent occlusion, 2) bleeding requiring blood transfusion, 3) fasting requiring for more than 7 days, and 4) any AE other than cholangitis caused by the procedure requiring additional intervention, including surgery. The remaining 15 patients were enrolled in Phase II. For the safety assessment, all patients underwent blood tests and abdominal computed tomography (CT) on the day after the EUS‐HGS procedure.

The type of stent used was not predetermined in this study. In principle, a fully covered self‐expandable metal stent (FCSEMS) was used; however, a plastic stent (PS) was placed in one patient. In this case, the puncture site was located near the B2/3 confluence, and placement of an FCSEMS was considered to carry a risk of segmental biliary obstruction and subsequent segmental cholangitis; therefore, PS was selected.

### 2.1 Endpoints

The primary endpoint was safety. The secondary endpoints were technical success rate, clinical success rate, re‐intervention methods, and frequency. We defined AEs occurring after the procedure and potentially related to it according to the American Society for Gastrointestinal Endoscopy lexicon [8]. AEs were defined as those occurring within 2 weeks of the procedure. Technical success was defined as the placement of a stent in the target bile duct, and clinical success was defined as improvement of cholangitis and/or ≥50% reduction in bilirubin within 30 days. Cholangitis was evaluated in terms of its occurrence and severity [9, 10].

### 2.2 Ethical Statement

This study was approved by the Institutional Review Board of the Aichi Cancer Center (approval number: IR2020‐1‐178) and was conducted in accordance with the Declaration of Helsinki. This study was registered with the University Hospital Medical Information Network Clinical Trials Registry (UMIN‐CTR; UMIN000047702).

### 2.3 EUS‐HGS Procedures

All procedures were performed under conscious sedation with intravenous administration of prophylactic antibiotics. EUS was performed using an oblique‐viewing echoendoscope (EG‐740UT; FUJIFILM Medical, Tokyo, Japan, or GF‐UCT260; Olympus, Tokyo, Japan) or forward‐viewing EUS (TGF‐UCT260J; Olympus). First, a landmark clip was positioned at the esophagogastric junction to aid in fluoroscopic visualization and minimize the risk of esophageal puncture [11]. A 22‐gauge needle (Expect Slimline; Boston Scientific) was loaded in advance with a 0.018‐inch guidewire (Fielder 18; Olympus) through a connector (Rotating Hemostatic Valve 0.096; Abbott, Tokyo, Japan) and filled with the contrast medium. A 19‐gauge needle (EZ Shot 3 Plus; Olympus) was loaded in advance with a 0.025‐inch guidewire (M‐through; Asahi Intecc, Aichi, Japan, or VisiGlide 2; Olympus) through a connector (Radifocus Hemostasis Valve II; Terumo, Tokyo, Japan) and filled with contrast medium. Color Doppler US guidance was used to avoid intervening vessels when puncturing the intrahepatic bile duct (IHBD). After inserting a guidewire into the B2 or B3 IHBD, a small volume of the contrast medium was injected. In cases where a 22‐gauge needle was used for puncture, the tract was dilated using a drill dilator (Tornus ES; Asahi Intecc, Aichi, Japan), followed by advancement of a catheter into the IHBD to inject contrast medium and aspirate bile. In cases punctured with a 19‐gauge needle, the catheter was directly advanced into the IHBD without tract dilation, and contrast injection and bile aspiration were performed. This technique served as the standard approach, and additional dilation was performed using a drill dilator or balloon catheter (REN; KANEKA, Osaka, Japan) as needed. Subsequently, an FCSEMS (6 mm × 12 cm HANAROSTENT Benefit; Boston Scientific, Boston, MA, USA, or Bile Rush covered Advanced 8 mm × 10 or 12 cm) or PS (7 Fr × 14 cm Through & Pass Type IT; Gadelius Medical, Tokyo, Japan) was placed. In some cases, bridging to the right IHBD was performed via the HGS route followed by stent placement. In these cases, the guidewire was advanced into the right IHBD, and the tract was dilated using a balloon catheter (REN; KANEKA, Osaka, Japan). An uncovered self‐expandable metal stent (UCSEMS) (8 mm × 6 cm Zilver 635 Biliary Self‐Expanding Stent; Cook Medical, Bloomington, USA) was deployed across the bridging route, and FCSEMS (6 mm × 12 cm HANAROSTENT Benefit; Boston Scientific, Boston, MA, USA) was placed along the HGS route. Bridging was performed when the right hepatic duct was opacified during EUS‐HGS. In this study, EUS‐HGS was first performed with additional drainage if necessary.

### 2.4 Statistical Analyses

The sample size of this study was determined based on exploratory and feasibility considerations, with safety assessment as the primary objective. All statistical analyses were performed using the JMP version 17.0 software (SAS Institute Inc., Cary, North Carolina, USA), with statistical significance set at *p* < 0.05. Overall survival (OS) was evaluated using the Kaplan–Meier method. Time to recurrent biliary obstruction (TRBO) was analyzed using a competing‐risk approach with death treated as a competing event. The cumulative incidence of TRBO was estimated using the cumulative incidence function.

## 3 Results

This study included 20 patients who consented to participate between June 2021 and December 2023. Patient characteristics are shown in Table 1. The bismuth classification of stenosis was I for four cases (20.0%), II for one case (5.0%), IIIa for five cases (25.0%), IIIb for three cases (15.0%), and IV for seven cases (35.0%). Cholangitis at the time of EUS‐HGS was observed in 15 (75.0%) patients. The severity was Grade I in seven patients (35.0%), Grade II in eight cases (40.0%), and Grade III in 0 patients. Ascites was observed in six patients (30.0%), and antithrombotic medications were routinely used in two patients (10.0%).

**TABLE 1:** Patient characteristics.

No. of cases	Phase I *n* = 5	Phase II *n* = 15
Age (years), median [range]	61.0 [38–72]	62.0 [47–82]
Sex (%)		
Male	3 (15.0)	8 (40.0)
Female	2 (10.0)	7 (35.0)
Primary disease (%)		
Gallbladder cancer	1 (5.0)	4 (20.0)
Intrahepatic bile duct cancer	1 (5.0)	3 (15.0)
Colorectal cancer	1 (5.0)	3 (15.0)
Pancreatic cancer	0 (0.0)	2 (10.0)
Gastric cancer	0 (0.0)	2 (10.0)
Hepatocellular cancer	0 (0.0)	1 (5.0)
Duodenal cancer	1 (5.0)	0 (0.0)
Kidney cancer	0 (0.0)	1 (5.0)
Bismuth classification (%)		
Type I	0 (0.0)	4 (20.0)
Type II	0 (0.0)	1 (5.0)
Type IIIa	2 (10.0)	3 (15.0)
Type IIIb	1 (5.0)	2 (10.0)
Type IV	3 (15.0)	4 (20.0)
Cholangitis status at the time of EUS‐HGS (%)	4 (20.0)	11 (55.0)
Absence	1 (5.0)	4 (20.0)
Presence	4 (15.0)	11 (55.0)
Grade I	2 (10.0)	5 (25.0)
Grade II	2 (10.0)	6 (30.0)
Grade III	0 (0.0)	0 (0.0)
Ascites (%)	2 (10.0)	4 (20.0)
mild	1 (5.0)	1 (5.0)
moderate	1 (5.0)	3 (15.0)
severe	0 (0.0)	0 (0.0)
Antithrombotic medications (%)	0 (0.0)	2 (10.0)

EUS‐HGS, Endoscopic ultrasound‐guided hepaticogastrostomy.

### 3.1 Outcomes of EUS‐HGS

**FIGURE 1:**
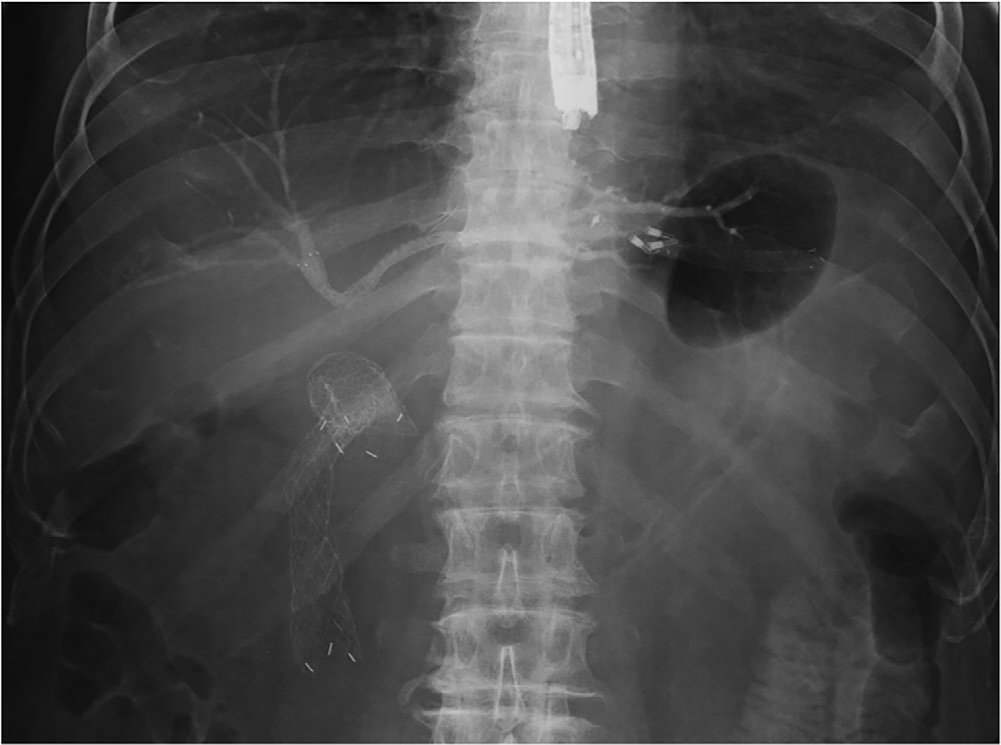
Procedure for bilateral drainage in patients with unresectable MHBO, in which primary EUS‐HGS is performed to create an HGS route, followed by the establishment of a bridging route to the right hepatic lobe through the HGS route. EUS‐HGS, endoscopic ultrasound‐guided hepaticogastrostomy; MHBO, malignant hilar biliary obstruction.

Table 2 shows details of the EUS‐HGS procedure. Procedure time (min), median [range] was 16.5 [8.0–64.0], and bile duct diameter (mm), median (range) was 4.1 [2.3–10.6]. Drainage method was B2 EUS‐HGS in 12 cases (60.0%), B3 EUS‐HGS in four cases (20.0%), B2 and B3 EUS‐HGS in two cases (10.0%), B2 EUS‐HGS + Bridging in one case (5.0%), and B3 EUS‐HGS + Bridging in one case (5.0%) (Figure 1).

**TABLE 2:** Details of the endoscopic ultrasound‐guided hepaticogastrostomy (EUS‐HGS) procedure.

No. of cases	Phase I *n* = 5 Five EUS‐HGS	Phase II *n* = 15 Seventeen EUS‐HGS
Procedure time (min), median [range]	13.0 [10.0–47.0]	15.0 [8.0–64.0]
Bile duct diameter (mm), median (range)	4.5 [2.4–5.8]	4.1 [2.3–10.6]
Needle‐gauge (%)		
22G	5 (25.0)	8 (40.0)
19G	0 (0.0)	6 (30.0)
19G ⇒ 22G	0 (0.0)	1 (5.0)
Puncture site (%)		
B2	4 (20.0)	9 (25.0)
B3	1 (5.0)	4 (10.0)
B2, B3	0 (0.0)	2 (10.0)
Drainage method (%)		
B2 EUS‐HGS Only	3 (15.0)	9 (45.0)
B3 EUS‐HGS Only	1 (5.0)	3 (15.0)
Both B2 and B3 EUS‐HGS	0 (0.0)	2 (10.0)
B2 EUS‐HGS + Bridging to the Right Lobe	1 (5.0)	0 (0.0)
B3 EUS‐HGS + Bridging to the Right Lobe	0 (0.0)	1 (5.0)
Fistula dilator device at EUS‐HGS route (%)		
ES dilator	3 (13.6)	0 (0.0)
Endosheather	0 (0.0)	1 (4.5)
Tornus	1 (4.5)	10 (45.5)
Balloon catheter	0 (0.0)	0 (0.0)
Electric cautery	0 (0.0)	0 (0.0)
Type of stent at EUS‐HGS route (%)		
HANAROSTENT Biliary Full Cover Benefit 6 mm × 12 cm	2 (9.1)	16 (72.7)
Covered BileRush Advanced 8 mm × 12 cm	2 (9.1)	1 (4.5)
Covered BileRush Advanced 8 mm × 10 cm	1 (4.5)	0 (0.0)
Through and Pass Type IT 7Fr 14cm	0 (0.0)	1 (4.5)
Fistula dilator device at the bridging route REN 6 mm + Uneven double lumen cannula	1	1
Type of stent at Bridging route Zilver 635 Biliary self‐expanding stent 8 mm × 6 cm	1	1

EUS‐HGS, Endoscopic ultrasound‐guided hepaticogastrostomy; PS, plastic stent; SEMS, Self‐expandable metallic stent.

Table 3 shows the outcome of EUS‐HGS. Technical success rate; 100% (20/20), clinical success rate; 75% (15/20). The four cases in which clinical success was not achieved were Bismuth type IV with cholangitis and obstructive jaundice, and all cases required additional drainage for the right bile duct. In addition, chemotherapy could be resumed after EUS‐HGS in 14 patients (70.0%).

**TABLE 3:** Outcomes of endoscopic ultrasound‐guided hepaticogastrostomy (EUS‐HGS).

No. of cases	Phase I *n* = 5	Phase II *n* = 15
Technical success rate, % (*n*/*N*)	100.0 (5/5)	100.0 (10/10)
Clinical success rate, % (*n*/*N*)	80.0 (4/5)	73.3 (11/15)
Early adverse events, % (*n*/*N*) ^*^	0.0 (0/0)	13.3 (2/15)
Stent migration	0	1, severe
Peritonitis	0	1, mild
Pancreatitis	0	0
Cholecystitis	0	0
Rate of chemotherapy administered after EUS‐HGS, % (*n*/*N*)	80.0 (4/5)	66.7 (10/15)

* Cotton et al. *Gastrointest Endosc*. 2010; 71: 446–54.

EUS‐HGS, endoscopic ultrasound‐guided hepaticogastrostomy.

**FIGURE 2:**
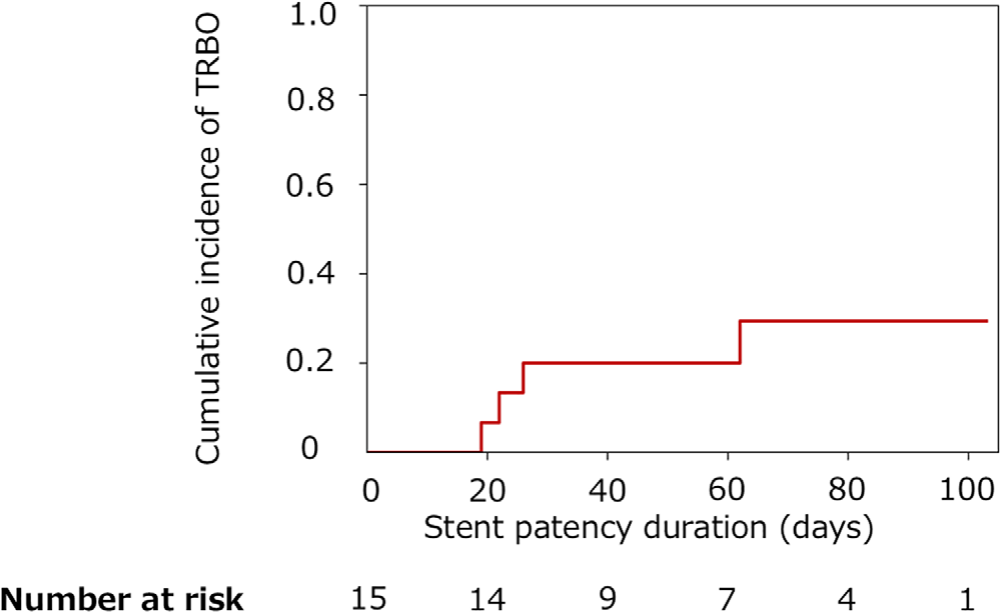
Cumulative incidence of TRBO after endoscopic ultrasound‐guided hepaticogastrostomy (EUS‐HGS). TRBO was defined as stent dysfunction requiring reintervention. Death was treated as a competing event. TRBO, time to recurrent biliary obstruction.

The median TRBO in patients who achieved clinical success, estimated using the Kaplan–Meier method, was 83.0 days (95% confidence interval [CI], 26.0–103.0). Because death precludes the occurrence of TRBO, a competing risk analysis was additionally performed with death treated as a competing event. The cumulative incidence of TRBO was 29.4% (Figure 2).

In Phase I, no early AEs occurred, and none met the predefined stopping criteria; therefore, the study advanced to Phase II. During Phase II, two early AEs were observed, resulting in an overall incidence of 10% (2/20) in the entire cohort. The early AEs consisted of one case of stent migration (severe) and one case of peritonitis (mild).

**FIGURE 3:**
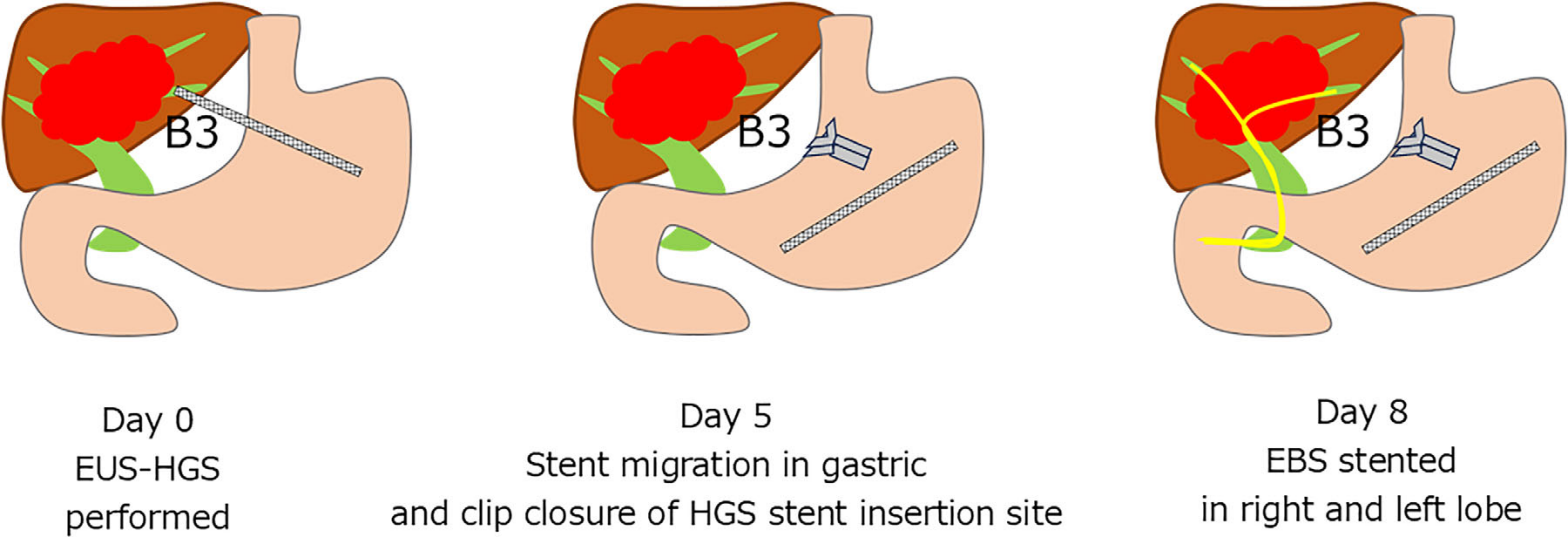
Clinical course of early severe stent migration after EUS‐HGS. EUS‐HGS was performed on Day 0. On Day 5, stent migration into the stomach was detected, and the HGS stent was removed, followed by endoscopic clip closure of the fistula site. On Day 8, EBS was performed for both the right and left hepatic lobes, resulting in adequate biliary drainage. EBS, endoscopic biliary stenting; EUS‐HGS, endoscopic ultrasound‐guided hepaticogastrostomy.

In this study, early stent migration (severe) occurred in one patient; therefore, the clinical course is described below. The patient with intrahepatic cholangiocarcinoma and peritoneal dissemination underwent chemotherapy. During the course, tumor progression led to obstructive jaundice, and EUS‐HGS was selected for primary biliary drainage. A 6 mm × 12 cm HANARO Benefit stent was placed into the B3 segment. Postoperative CT on the following day confirmed appropriate stent placement without early AEs, and oral intake was resumed. However, on day 5, the patient developed abdominal pain. CT revealed stent migration into the stomach. Endoscopy confirmed intragastric migration from the liver. The stent was removed, and the anastomosis was closed with endoscopic clips. Given the presence of increasing ascites on CT, intraperitoneal bile leakage was suspected, and a peritoneal drain was placed. The patient's symptoms subsequently improved. On day 8, endoscopic biliary stenting (EBS) was performed for both the anterior and lateral segments. Adequate biliary drainage was achieved thereafter (Figure 3).

**FIGURE 4:**
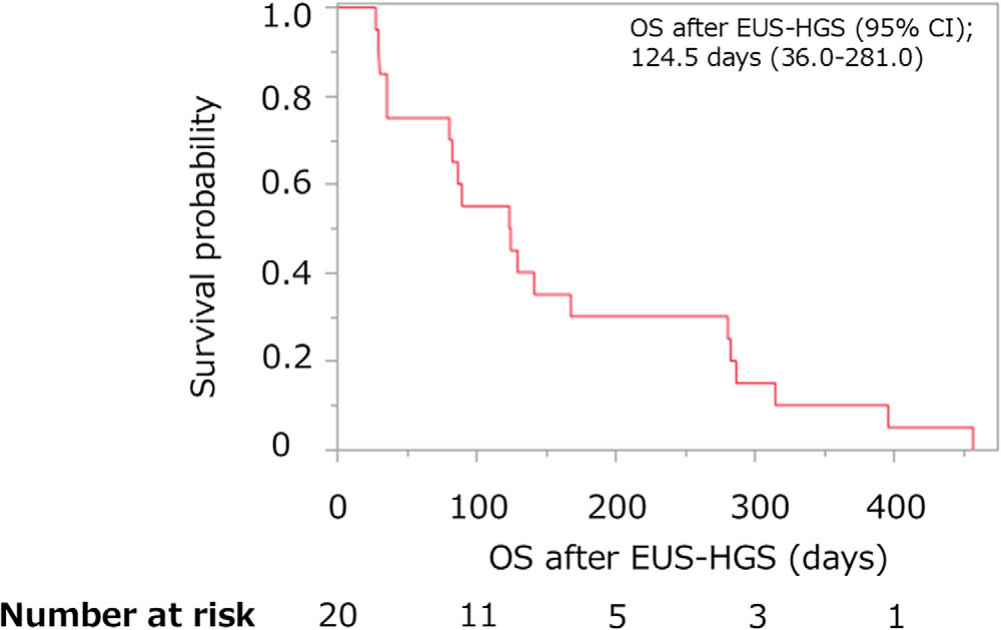
This figure shows Kaplan‐Meier curves of OS. The OS after endoscopic ultrasound‐guided hepaticogastrostomy (EUS‐HGS) (95% CI) was 124.5 days (36.0–281.0). CI, confidence interval; OS, Overall survival.

Table 4 shows the outcome of re‐intervention after EUS‐HGS. The success rate of re‐intervention was 100% (10/10). Scheduled stent exchange was performed in six patients. However, three patients developed cholangitis and required unscheduled stent exchange, one patient experienced stent migration necessitating stent reinsertion, in another patient, although stent migration was observed, no elevation of hepatobiliary enzymes or biliary dilatation occurred during chemotherapy, and therefore additional biliary drainage or stent reintervention was not considered necessary. Number of re‐interventions, median [range] was 3 [1–4]. The right lobe drainage method with additional re‐intervention was 50% (5/10). Specifically, there were three cases of EBS and two cases of bridging. The OS after EUS‐HGS was 124.5 days (95% CI, 36.0–281.0) (Figure 4).

**TABLE 4:** Outcomes of re‐intervention after endoscopic ultrasound‐guided hepaticogastrostomy (EUS‐HGS).

No. of cases	Phase I *n* = 3	Phase II *n* = 7
Number of re‐interventions, median [range]	1 [1–3]	3 [1–4]
Re‐intervention technical success rate, % (*n*/*N*)	100.0 (3/3)	100.0 (7/7)
Right lobe drainage method with additional re‐intervention, % (*n*/*N*)	66.7 (2/3)	42.9 (3/7)
EBS	2	1
Bridging	0	2

EBS, endoscopic biliary stent; EUS‐HGS, endoscopic ultrasound‐guided hepaticogastrostomy.

## 4 Discussion

Traditionally, transpapillary drainage has been performed for MHBO. Recently, additional EUS‐HGS has been performed in cases where transpapillary drainage alone is insufficient [4, 5, 6, 7]. In this study, we investigated the safety of EUS‐HGS as the primary drainage method for unresectable MHBO.

In this study, stent migration was observed in one case (severe) and peritonitis in one case (mild). Stent migration was considered to be caused by the presence of ascites along the puncture tract and the short intraductal length of the stent placed in the IHBD. Previous reports have indicated that the presence of ascites and a long distance between the stomach and the IHBD are risk factors for stent migration. The AEs observed in this case were consistent with those findings [12, 13]. When a stent is placed in the B3 segment, its intragastric position tends to be more distal than that in B2. Consequently, mechanical stimulation due to food intake may increase the risk of stent migration. Indeed, there have been reports of stent migration in cases in which an HGS stent was placed in B3 [14]. To prevent such AEs, we performed ascites drainage before EUS‐HGS, placed the stent with as long an intraductal length as possible within the IHBD, and avoided stent placement in the B3 segment whenever feasible [15]. No other AEs, such as pancreatitis and bleeding, or serious AEs were observed. Compared to previous reports, the incidence of AEs in this study was low, suggesting that EUS‐HGS is safe as the primary drainage method for unresectable MHBO [1, 16, 17, 18, 19, 20]. However, safety comparisons with ERCP should take into account the presence of ascites and the need for preprocedural drainage. Therefore, the safety of EUS‐HGS should be interpreted in the context of appropriate patient selection and procedural precautions rather than being generalized unconditionally.

EUS‐HGS has several advantages as a primary drainage procedure. First, EUS‐HGS can become technically challenging once ascites develops because of disease progression; therefore, securing a drainage route before the appearance of ascites is a significant benefit [21]. Second, by creating a drainage route that does not pass through the tumor, stable and easy reintervention becomes possible. Because reintervention can also be performed via the EUS‐HGS route when needed, EUS‐HGS is considered a highly useful primary drainage method.

In contrast, the clinical success rate was 75%. Table 5 presents the clinical success rates according to the bismuth classification system. According to this table, the clinical success rate for bismuth type IV was 42.9%. In all cases in which clinical success was not achieved, additional drainage of the right lobe was required. Therefore, when performing EUS‐HGS as the primary drainage method in patients with bismuth type IV tumors, simultaneous drainage of the right lobe is recommended, and further refinement is necessary.

**TABLE 5:** Clinical success rate of the bismuth classification.

	Clinical success rate, % (*n*/*N*)	
Bismuth classification	Phase I *n* = 5	Phase II *n* = 15
Type I	0.0 (0/0)	100.0 (4/4)
Type II	0.0 (0/0)	100.0 (1/1)
Type IIIa	100.0 (2/2)	66.7 (2/3)
Type IIIb	100.0 (1/1)	100.0 (2/2)
Type IV	50.0 (1/2)	42.9 (2/5)

This study has several limitations. First, it was conducted at a single center with a relatively small number of cases. However, as no previous prospective studies have evaluated EUS‐HGS as a primary drainage method, particularly for MHBO, this study is highly valuable. Second, the underlying diseases and stricture sites were not uniform among patients. Therefore, prospective randomized controlled trials with larger sample sizes are required to validate our findings.

In conclusion, the safety of EUS‐HGS for the primary drainage of unresectable MHBO is acceptable. Regarding clinical efficacy, based on the results observed in patients with Bismuth type IV obstruction, we acknowledge that further investigation is warranted.

## Author Contributions

All authors participated in conducting this research. **Tomoki Ogata**: manuscript writing, drafting conception, and design; **Kazuo Hara** and **Nozomi Okuno**: drafted, conceived, and designed the study, performed the endoscopic procedures, and assisted with manuscript writing; **Shin Haba**, **Takamichi Kuwahara**, **Shimpei Matsumoto**, **Hiroki Koda**, and **Keigo Oshiro**: performed endoscopic procedures and analyzed the data. All authors have approved the final draft of the manuscript.

## Conflicts of Interest

The authors declare a potential conflict of interest with Asahi Intecc, Aichi, Japan.

## Funding

The authors have nothing to report.

## Ethics Statement

**Approval of the research protocol by an Institutional Reviewer Board**: This study was approved by the Institutional Review Board of the Aichi Cancer Center (approval number: IR2020‐1‐178).

## Consent

Written informed consent was obtained from all participants prior to participation in the study.

## Data Availability

The datasets used and/or analyzed in the current study are available from the corresponding author upon reasonable request.
